# The Temporal Pattern, Flux, and Function of Autophagy in Spinal Cord Injury

**DOI:** 10.3390/ijms18020466

**Published:** 2017-02-21

**Authors:** Kailiang Zhou, Charles A. Sansur, Huazi Xu, Xiaofeng Jia

**Affiliations:** 1 Department of Orthopaedics, The Second Affiliated Hospital and Yuying Children’s Hospital of Wenzhou Medical University, Wenzhou 325027, China; zhoukailiang@wmu.edu.cn (K.Z.); spinexu@163.com (H.X.); 2 Department of Neurosurgery, University of Maryland School of Medicine, Baltimore, MD 21201, USA; CSansur@som.umaryland.edu; 3 Department of Orthopaedics, University of Maryland School of Medicine, Baltimore, MD 21201, USA; 4 Department of Anatomy and Neurobiology, University of Maryland School of Medicine, Baltimore, MD 21201, USA; 5 Department of Biomedical Engineering, The Johns Hopkins University School of Medicine, Baltimore, MD 21205, USA; 6 Department of Anesthesiology and Critical Care Medicine, The Johns Hopkins University School of Medicine, Baltimore, MD 21205, USA

**Keywords:** temporal pattern, flux, function, autophagy, traumatic spinal cord injury, ischemia-reperfusion spinal cord injury

## Abstract

Previous studies have indicated that autophagy plays a critical role in spinal cord injury (SCI), including traumatic spinal cord injury (TSCI) and ischemia-reperfusion spinal cord injury (IRSCI). However, while the understanding of mechanisms underlying autophagy in SCI has progressed, there remain several controversial points: (1) temporal pattern results of autophagic activation after SCI are not consistent across studies; (2) effect of accumulation of autophagosomes due to the blockade or enhancement of autophagic flux is uncertain; (3) overall effect of enhanced autophagy remains undefined, with both beneficial and detrimental outcomes reported in SCI literature. In this review, the temporal pattern of autophagic activation, autophagic flux, autophagic cell death, relationship between autophagy and apoptosis, and pharmacological intervention of autophagy in TSCI (contusion injury, compression injury and hemisection injury) and IRSCI are discussed. Types of SCI and severity appear to contribute to differences in outcomes regarding temporal pattern, flux, and function of autophagy. With future development of specific strategies on autophagy intervention, autophagy may play an important role in improving functional recovery in patients with SCI.

## 1. Introduction

Spinal cord injury (SCI) is a major disabling disease currently with insufficient treatment options. It not only impairs physical and psychological health, but imposes substantial financial burden on family and society. The lifetime cost of SCI for an individual injured at age 25 is estimated to exceed 2 million dollars in the United States [1]. According to different pathogenic factors, SCI is divided into traumatic spinal cord injury (TSCI) and non-traumatic spinal cord injury (NTSCI) [2]. TSCI is often caused by events the result in extrinsic compression of the spinal cord such as motor vehicle accidents (38% of cases), falls (>22%), violence (13.5%), and sports and recreational accidents (9%) [3]. In the United States alone, about 27,300 people are affected by TSCI, with almost 12,000 new cases occurring every year [4]. Damage to the spinal cord from pathology other than trauma has been referred to as non-traumatic spinal cord injury (NTSCI). Clinically, ischemia-reperfusion spinal cord injury (IRSCI) after clinical surgery is a common type of NTSCI, with incidence of 3%–18% [5]. In the clinical setting, descending thoracic or thoracoabdominal aortic aneurysm surgery, due to inherent need temporarily reduce blood flow in aorta, may result in postoperative IRSCI [6]. The histologic changes in TSCI are conventionally divided into primary injury phase and second injury phase. The immediate compression and disruption of axons and vasculature are results of primary injury (mechanical trauma) [7]. Then, a cascade of events of histology are triggered secondary to the injury, including edema, hemorrhage, inflammation, demyelination, neuronal and oligodendroglial changes, and microglial and astrocyte activation in early stage; Wallerian degeneration, scar formation, development of cysts and syrinx, and schwannosis in later stage [8]. The main histologic damages caused by IRSCI are involved in activation of microglial and astrocyte, blood–spinal cord barrier (BSCB) disruption, tissue edema and neutrophil influx after spinal cord ischemia [9]. Both TSCI and IRSCI can result in loss of motor function, impaired sensation, and severe damage to the autonomic nervous system.

**Figure 1:**
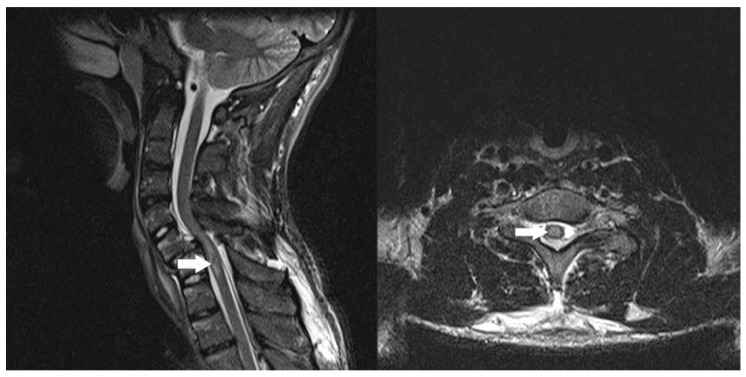
In spinal cord Magnetic Resonance Imaging (MRI) of patients who suffered traumatic spinal cord injury (TSCI), spinal cord hemorrhage and edema were characterized by increased T2-weighted signal within 72 h of injury. This non-contrast T2 weighted, short tau inversion recovery (STIR) sequence MRI depicts the sagittal and axial cross sections of a 17-year-old male 8 h after suffering from a ski accident. White arrows which depict increased T2 signal within the spinal cord indicate edema consistent with traumatic spinal cord injury (TSCI).

Strenuous efforts have been made to develop effective clinical management strategies in the setting of SCI. These include: surgical decompression of the spinal cord, pharmacologic cord protection (methylprednisolone sodium succinate), pluripotent stem cells application, hypothermia therapy for TSCI [10]; other surgical interventions (such as retrograde venous perfusion [11] and ischemic preconditioning [12]), and pharmacologic management (oxygen free radical scavengers [6], Ca^2+^ channel blockers [13] and methylprednisolone [14]) for IRSCI. Unfortunately, the recovery of motor function after the vast majority of SCIs is still not substantial. To develop more effective therapies, understanding the mechanisms and pathology involved in SCI is critical. However, its pathophysiology and mechanisms involved in progression remain unknown. After mechanical trauma impact to the spine, resulting in the immediate compression, contusion (Figure 1), incision, and stretching or kinking of the spinal cord, damage is incurred on axons, blood vessels and neurons, including cellular strain and plasma membrane damage [15]. This environment triggers a secondary cascade of events including ischemia and excitotoxic chemical release, which accelerate local neural cell death and expand the lesion [7]. Then, cavity is formed and infiltrated by inflammatory cells, microglia, fibroblasts and reactive astrocytes [16]. Finally, fibrous glial scar formation, Wallerian degeneration and chronic demyelination occur, and potential therapy is limited over time [17].

Spinal cord MRI of patients with ischemia-reperfusion spinal cord injury (IRSCI) reveals enlargement of the spinal cord with increased T2 weighted signal 1–2 days after injury. After 2 days, spinal cord enhancement is present with administration of gadolinium contrast [18]. Pathophysiology of acute changes triggered in IRSCI is primary involved in these events: over-produced reactive oxygen species (ROS) from the damaged mitochondria results in neuronal cells death through protein breakdown, lipid peroxidation, and DNA damage [19]. Furthermore, developed oxidative stress status in the lesion triggers microglial and astrocyte activation and releases excessive proinflammatory mediators that promote blood–spinal cord barrier (BSCB) disruption and neutrophil influx, and contribute to inflammation and subsequent neural cell death after spinal cord ischemia [20]. Neural cell death and loss contribute to pathologic process and further functional deficiency after spinal cord injury (SCI), thus, blocking cells death in SCI may provide a novel therapeutic approach for neuroprotection.

Macroautophagy (hereafter named autophagy), a major pathway for bulk cytosolic degradation and efficient turnover under stress, has an important role in maintaining cellular homeostasis by degrading and recycling damaged organelles, toxic agents and long-lived, unwanted proteins through an autophagosomal–lysosomal pathway [21]. The autophagic process is carried out in three steps: formation of autophagosomes, fusion of autophagosomes with lysosomes, and degradation in the autolysosome [22]. Growing evidence has shown that autophagy can act as a pro-survival mechanism via regulating the neural cells death for neuroprotection in central nervous system (CNS) diseases [23], particularly in SCI [24,25]. Among various neural cells, neurons are the neural cell type most commonly reported to activate autophagy and benefit from it, in studies of SCI [26,27]. The pathologic mechanism is that autophagy depresses neuronal cell death and loss via promoting the elimination of toxic proteins and damaged mitochondria (mitophagy) [28,29,30]. However, other neural cell types, astrocytes, oligodendrocytes and microglia, in SCI presented upregulation of autophagy [27,31,32], but functions of autophagy in these cells after SCI are unclear currently.

**Figure 2:**
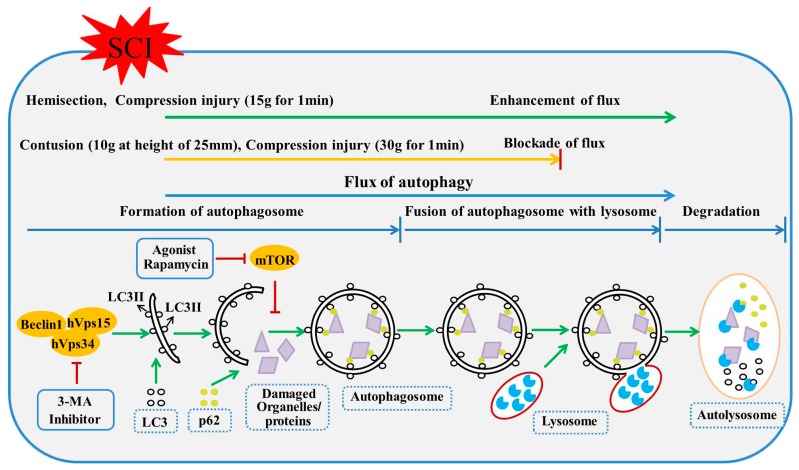
The autophagic process includes three steps: formation of autophagosomes, fusion of autophagosomes with lysosomes, and degradation in the autolysosome. Flux of autophagy is used to describe the whole dynamic process of autophagy, and is discrepant in different traumatic spinal cord injury (TSCI) types. The flux is enhanced in hemisection injury [27] and moderate compression injury (15 g for 1 min) [26], whereas it is blocked in severe contusion injury (10 g at height of 25 mm) [32] and compression injury (30 g for 1 min) [38]. Autophagic agonists (rapamycin) and inhibitors (3-methyladenine, 3-MA) targeting the process of autophagy are presented. The green arrows stands for progressing autophagy process and red T-bars stands for blocking of autophagy process. LC3: microtubule-associated 1 protein light chain 3; mTOR: mammalian target of rapamycin.

Several biological markers have been developed to characterize the process of cell autophagy in past years. Among them, microtubule-associated 1 protein light chain 3 (LC3), Beclin1 and p62 are commonly used to monitor activity of cell autophagy in SCI. LC3 proteins, located on the membrane surface of pre-autophagic vacuoles and autophagic vacuoles, are involved in autophagosome formation [33]. There are two types of LC3 proteins: LC3I and LC3II. When autophagy occurs, LC3I undergoes ubiquitin-like changes and binds to phosphatidylethanolamine (PE) on the surface of vacuole membranes of the autophagosome, resulting in the formation of LC3II which is bound to the membrane of autophagic vacuoles [34] (Figure 2). Therefore, LC3II expression or the ratio of LC3II/LC3I is a straightforward index used to reflect the number of autophagic vacuoles [35]. In addition, one of the other autophagic vacuoles markers is the Beclin1 protein. The Beclin1-Vacuolar protein sorting34-Vacuolar protein sorting15 (Vps34-Vps15) core complex is required in the pre-autophagosomal structure, and hence the expression of Beclin1 correlates closely to autophagosome activity [36] (Figure 2). Since autophagy is a dynamic mechanism to degrade damaged cellular organelles and unwanted proteins, it is useful to develop and test degradation markers. For example, p62 (also known as Sequestosome 1 (SQSTM1)/sequestome1) is incorporated into autophagosomes through binding to LC3 and subsequently degrades through autophagy, hence p62 protein levels can be used to assess autophagic degradation [37] (Figure 2).

In the autophagic studies of SCI, Kanno and coworkers were the first to report that autophagic proteins Beclin1 and LC3II were overexpressed around the lesion of experimental TSCI [31,39]. In 2009, Baba et al. found that LC3II was highly expressed in the IRSCI rabbit model [40]. Since then, many studies have focused on the mechanisms and effects of autophagy on both TSCI and IRSCI pathologic processes [25,30,41,42]. Though it is widely accepted that autophagy plays a critical role in spinal cord injuries, recent exploration and studies revealed several controversial points: (1) temporal pattern results of autophagic activation after SCI are not consistent across studies; (2) effect of accumulation of autophagosomes due to the blockade or enhancement of autophagic flux is uncertain; and (3) overall effect of increased autophagy remains undefined, with both beneficial and detrimental outcomes reported in literature. This review will summarize findings of current studies in order to identify temporal pattern, flux, and function of autophagy in both TSCI and IRSCI.

## 2. Temporal Pattern of Autophagic Activation in SCI

Studies about the temporal pattern of autophagic activation in SCI are fundamental and crucial. They are beneficial for understanding progress of autophagy and its relationships with other pathologic events, and choosing appropriate time points for autophagic regulation after SCI. However, results from these studies are not consistent. Different temporal patterns of autophagic activation between were presented for different SCI types. These results are represented for TSCI and IRSCI as follows.

### 2.1. Temporal Pattern of Autophagy in TSCI

TSCI may be induced by various mechanical traumas to spine and spinal cord, and can be classified by different mechanical injuries such as transection injuries, contusion injuries, compression injuries, distraction injuries, dislocation injuries etc. [43]. In experimental TSCI, rodent models are used routinely to simulate various types of TSCI. Among these models, hemisection injury, contusion injury, and compression injury are most frequently used, especially in the autophagic SCI experimental research [27,28,44].

#### 2.1.1. Spinal Cord Hemisection Injury

In 2008, the first experimental study of autophagy following SCI was performed in mice by Kanno et al. using a hemisection injury. The exposed spinal cord was cut with a sharp scalpel on the right side of the spinal cord to create a hemisection injury [39]. After “primary injury”, the number of the Beclin1 positive cells in the lesion increased at 4 h, peaked at 3 days, and then gradually decreased to near normal levels 21-days post injury. The Beclin1 positive cells were observed in neurons, astrocytes, and oligodendrocytes [31]. Two years later, they found similar results of autophagy via labeling LC3 proteins with similar temporal pattern of LC3 positive cells in the same mouse model [39]. Subsequently, Tang et al. presented the similar activated autophagy trends in similar time points via western bolt analysis for LC3II in a rat hemisection SCI model [27,45].

#### 2.1.2. Spinal Cord Contusion Injury

Approximately half of SCI cases seen in the clinical realm result from contusion injury [8]. Therefore, spinal cord contusion injury is commonly modeled in experimental research. A modified New York University impactor [46,47,48] was used to mimic the transient force applied to the spinal cord for the creation of a contusion. The spinal cord in rats or mice were exposed by laminectomy, and then impacted by dropping a rod with a specific weight from a specific height [25]. The severity of contusion depends on the rod weight and dropping height. It is consistently acceptable that dropping a 10 g weight from the height of 25 mm induced a severe degree of SCI in the rat model [49], which is commonly used in the autophagic study of spinal cord contusion [32,50,51,52,53]. Using this severe contusion injury model, past studies showed that autophagy was present at higher levels when compared to the non-injured case at single time points after SCI, namely, at day 1 [54], day 3 [44,55], day 4 [56], and day 7 [57] after injury. However, the temporal pattern analysis that reveals changes in autophagic activity over time were not investigated in these studies. Fortunately, two research teams have given further insight on this matter. Autophagic temporal pattern results in contusion injuries seem to be influenced by the degree of contusion to spinal cord. Hao et al. demonstrated that the expression levels of Beclin1 and LC3II in lesion started to decrease at 1 h, peaked at 2 h, and gradually decreased to near normal levels at 24 h in animals with severe contusion injuries (10 g at height of 25 mm) [51]. In the relatively moderate spinal cord contusion (8 g at height of 25 mm), different autophagic temporal trends have been reported. The ratio of LC3II/I in the injured spinal cord decreased at 3 days, peaked at 10 days, and finally increased to the baseline at 21 days after SCI [58].

#### 2.1.3. Spinal Cord Compression Injury

Compression models are used to induce persistent spinal cord pressure, which is commonly seen in human TSCI, and also to investigate the effect of compression and/or the optimal time of decompression after injury [59]. This model is frequently applied to examine autophagy in the spinal cord lesions created by compression using the clip compression model. The clip compression method requires spinal cord exposure and application of vascular clip, which produces a certain amount of force and is dorsoventrally closed over the entire spinal cord for a specific amount of time. In autophagic studies of compression injury, many articles report that the ratio of LC3II/I or Beclin1 expression is significantly increased in the lesion at 3 and 7 days after SCI [26,38,60]. However, these studies only investigated single time points. Further investigation is needed to elucidate the temporal pattern and change of autophagic activity over multiple time points after injury.

### 2.2. Temporal Pattern of Autophagy in IRSCI

The concept of IRSCI is that reperfusion of ischemic spinal cord does not improve the neurological function of the injured spinal cord, but further aggravates it, and even results in the irreversible delayed neuronal death of spinal cord [61]. The pathologic process of IRSCI is divided into an ischemic period and reperfusion period. The duration of the ischemic period plays a critical role for functional recovery after IRSCI [62]. Recently, researchers have focused on the temporal patterns of autophagic activity in IRSCI, but the results have been inconsistent. In a previous study, rats received thoracic aortic arch exposure and clamping for 14 min to establish the spinal ischemia/reperfusion (I/R) injury model [63]. After spinal cord ischemia, both the ratio of LC3II/LC3I and Beclin1 expression increased and peaked twice, once at 8 h (early stage) and once at 72 h (late stage), and then slowly reduced to baseline. However, the different results of autophagic temporal pattern have also been reported, and seem to be influenced by the duration of ischemia. In the study of Wei et al., 10-min ischemia of the thoracic aortic arch was performed via balloon inflation, and autophagic assessments were performed after spinal cord ischemia in a time dependent manner [64]. The results showed that LC3II and Beclin1 expressions combined with LC3II positive cells in lesion started to increase at 3 h, peaked at 24 h, and maintained a high level at 48 h after the ischemia [64], however, decline to normal and increases to other peaks were unknown.

These results indicate that different injury types or injury severity differentially affect temporal pattern of autophagic activation in SCI. In another common neurotraumatic disease, traumatic brain injury (TBI), it was suggested that severities of injury may be a key determinant of activation and process of autophagy [25]. The potential underlying mechanism may be that different severity of injury in TBI influences the capacity of cells near the injury location to formation and degrades autophagosomes, which leads to the difference in autopohagic procession [65]. According to this hypothesis, it is feasible to explain the phenomenon that temporal patterns of autophagy are varied in different in severities of injury in the same type of SCI, which is found in contusion injury and I/R injury, respectively.

Although there is no quantitative index to evaluate the severity between different SCI types mentioned above, it is clear that the condition of lesion after primary injury is inconsistent after these SCIs. For instance, in hemisection the injured tissue is focused at the epicenter of injury (white matter apoptosis, demyelination and microglia activation at the epicenter); contusion injury has widespread tissue pathology, extending both rostrally and caudally from epicenter of injury (disruption of white and gray matter, intraparenchymal hemorrhage, diffuse axonal injury and activated microglial at epicenter); compression injury induces significant vascular injury (hemorrhagic necrosis and hypoperfusion) and neuronal ischemia; and ischemia–reperfusion injury mainly focuses on vascular derangement (ischemia, hypoxia, vasospasms, thrombosis) [43]. These different pathologic conditions of SCIs may result in various severities of damage to the cord, and the autophagy in the injury nearby cells may be activated to protect against stress events in different velocities and intensities.

## 3. Autophagic Flux Blockade or Enhancement in SCI

The term “autophagic flux” has been used to describe the autophagy as a dynamic process, encompassing the entire process of autophagy holistically. This would include autophagosome formation with subsequent maturation followed by fusion with lysosome, breakdown, and dispensing of toxic proteins and damaged organelles back into the cytosol [66] (Figure 2). Autophagic flux is important for intracellular “refreshing”, and this role in homeostasis is particularly crucial for the health of terminally differentiated cells such as neurons and oligodendrocytes [67,68]. Increasing literature reported that blocking of autophagic flux in neurons results from lysosome defects or the failure in fusion between atophagosomes and lysosomes, and leads to the development of some CNS diseases [23,69]. Particularly, neurodegenerative diseases, like Parkinson’s, Alzheimer’s, and Huntington’s disease, are associated with autophagy defect, leading to accumulation of ubiquitin-positive protein aggregates and subsequent neuronal cell death [70,71,72]. However, relatively fewer SCI studies have focused on the state of autophagic flux and its role compared to the number of studies on autophagosomes. It is widely accepted that increase of autophagosomes results from two situations. One is that the increased autophagosomes are triggered for fusion with lysosomes, which indicates autophagic flux enhancement. The other is that the incremental autophagosomes are accumulated through an interdiction in fusion of autophagosomes with lysosomes, which represents autophagic flux blockade. However, many studies suggest that increased autophagosomes could indicate the enhanced dynamic process of autophagy after both TSCI and IRSCI [35,42,44,53,54,73]. This may represent a blockade in fusion of autophagosome with lysosome.

To test the flux of autophagy, three classical methods have been developed to provide direct evidence regarding autophagic substrate degradation in lysosomes. These include assays based on dynamic LC3 turnover, degradation of p62, and degradation of long-lived proteins [66]. Among these methods, the testing of p62 degradation via western blot analysis or immunofluorescence is relatively convenient, and is widely applied to track autophagic flux following various diseases, including SCI [32]. p62 serves as a special maker for assessing the degradation of autophagy, which can reflect autophagic flux. For instance, autophagic flux blockade correlates with an increased p62 level, and autophagic flux enhancement correlates with a decreased p62 level [74]. The situation of autophagic flux in hemisection injury [27], contusion injury [32] and compression injury [75] has been studied, but not in ischemia-reperfusion injury [12]. Expressions of p62 were different in hemisection injury, contusion injury and compression injury. In the spinal hemisection injury model, levels of p62 declined at 4 h after SCI, reaching the lowest point at 3 days and lasting for at least 21 days [45]. This result demonstrated that autophagic flux is activated after spinal cord hemisection injury (Figure 2). Currently, the spinal cord contusion model for autophagic flux evaluation is only induced by severe contusion (10 g at height of 25 mm). In this spinal cord contusion model, the level of p62 increased immediately after injury, with a peak at day 1, then decreased by day 7 but remained above baseline for at least 5 weeks after contusion injury [68]. This indicates that autophagic flux was compromised after severe spinal cord contusion injury (Figure 2). However, the results in compression injury were diverse, and seem to be due to the different injury severities. The vascular clip compression method is usually used to induce the spinal cord compression injury model in rats. The injury severity of compression depends on force of vascular clip and time of the compression. Previous studies have compressed the spinal cord with three different clips with different closing force (3, 8, and 24 g) for 1 min to induce the mild, moderate and severe SCI model, respectively [76]. Zhang et al. demonstrated that the expression level of p62 in lesion was increased at day 3 after the severe compression injury induced by vascular clip (30 g force) compression for 1 min to the spinal cord [38]. However, in our previous studies, the increased expression of p62 was found in injured spinal cord at day 7 after relatively moderate compression injury (15 g for 1 min) [38,77]. According to the current results, severe compression injury may contribute to autophagic flux blockade, and moderate compression injury may result in autophagic flux enhancement. Considering these two studies’ p62 evaluations at different time points, further studies at the same time points are necessary.

In summary, the progress of autophagic flux is varied in different types of SCI. The flux of autophagy is blocked in severe contusion injury (10 g at height of 25 mm), and enhanced in hemisection injury. In compression injury, the results of autophagic flux are not consistent. It is inhibited after severe compression (15 g for 1 min), and enhanced after relatively moderate compression (30 g for 1 min). Reasons for these varying results of autophagic flux across studies are unclear. It is possible that injury severity may differentially affect the completion of autophagic flux in SCI. The mechanism underlying the explanation of these results still requires further research. A similar result of the interaction of injury severity and autophagic flux was found in TBI. Namely, autophagic flux is increased after moderate injury, and decreased after severe trauma, at the lesion of TBI [25]. The potential mechanism suggested was that the severe injury could alter the ability of neural cells near the injury location to correctly deliver and degrade autophagosomal cargo, finally resulting in blocking of autophagic flux [65]. According to this hypothesis, it is reasonable to explain that flux of autophagy is blocked in severe SCIs, contusion injury (10 g at height of 25 mm) and compression injury (30 g for 1 min), and enhanced in moderate compression injury (15 g for 1 min). For hemisection injury, the tissue injury is focused on the epicenter of injury, therefore nearby issue and neural cells of injury area are less damaged. Thus, hemisection injury just causes a half injury in the cross section and might be mild or moderate, which increases flux of autophagy. The detail mechanism underlying the explanation of these results still requires further research.

## 4. Autophagic Cell Death in SCI

Cell death occurs by various mechanisms, including deliberate suicide of an unwanted cell in a multicellular organism, known as cell programmed cell death (PCD) [78]. Cell apoptosis is the most known PCD mechanism [79]. Recently, another type of PCD, termed autophagic cell death (ACD), plays a significant role non-apoptotic programmed cell death [80]. ACD is mainly a morphologic definition, which is characterized by a high level of autophagosomes and features of active autophagy in dying cells [81]. Whether intense autophagy in ACD leads the execution of cell death or is an attempt at cell-survival from PCD remains highly controversial [79].

Previous studies have found that ACD occurs in various diseases, like cerebral ischemia, traumatic brain injury, renal ischemia and reperfusion injury [82,83,84]. In some experimental SCI studies, instead of cytoprotection, worse outcomes were found after the pharmacological stimulation of autophagy; therefore, ACD was considered [28,63]. However, the molecular marker to define the presence of autophagic cell death is still not well understood. In addition to the presence of intense autophagy, ACD has a distinct nuclear morphology distinct from that of apoptosis [80]. Apoptosis is characterized by cellular shrinkage with condensation of the cytoplasm, sharp delineation of chromatin masses lying against the nuclear membrane, nuclear fragmentation, and subsequent formation of membrane-confined apoptotic bodies [85]. In ACD, the nucleus does not shrink or get fragmented, and there is increased formation of autophagic vacuoles [83]. Upon this theoretical basis, the double staining of LC3 and transferase-mediated dUTP nick end labeling (TUNEL) is performed by researchers to characterize ACD by identifying DNA fragmentation in the cells expressing LC3. Kanno et al. found round nuclei in cells that were TUNEL-positive and expressed LC3, which supports ACD in the model of hemisection injury [39]. However, the role of activated autophagy in ACD is not defined in this study. On the other hand, in the same model, beneficial outcomes were reported after the exogenous stimulation of autophagy [27]. This indicates that the process of autophagy in ACD may be a protective mechanism to survive from cell death after hemisection injury. Whether ACD occurs and plays a similar role in other types of SCI remains to be investigated.

## 5. Relationship between Autophagy and Apoptosis after SCI

It is widely accepted that apoptosis plays an important role in cell death and axon disruption after SCI. Therapeutic actions targeting anti-apoptosis resulted in better neurological outcomes in experimental SCI. Increasing amounts of literature report that there is a close biochemical crosstalk mechanism between apoptosis and autophagy [86]. There are potentially many common signal transduction pathways that influence both autophagy and apoptosis such as p53 protein, Bcl-2-homology-3-only (BH3‑only) proteins, Ser/Thr kinases, oncogenes and so on, and these processes demonstrate the ability for autophagy and apoptosis to cross-regulate each other through inhibition [87]. In autophagic studies of TSCI and IRSCI, such inhibitory crosstalk was found, and stimulation of autophagy significantly decreased the level of apoptosis in neurons, thus a neuroprotection and functional recovery was reported [26,27,44,63].

The mechanism through which autophagy reduces the tendency of cells to lead to apoptosis is multitudinous, and one of the principal mechanisms is autophagic flux blockade [88]. The disruption of autophagic flux after SCI exacerbates ER stress and contributes to ER stress-induced cell apoptosis [89]. When autophagic flux is blocked, the level of apoptosis increases in contusion injury and severe compression injury [32,38]. Furthermore, increasing the level of autophagic flux can therefore inhibit apoptosis in those SCI models [38,44]. However, this mechanism does not seem to explain the inhibition of apoptosis by autophagy in hemisection injury [27] and moderate compression injury [77], where the flux of autophagy was enhanced. It is important to note that mitophagy (autophagy of mitochondria) also plays a role in inhibition of apoptosis by autophagy in various diseases [90,91]. The dysfunction of mitochondrial can trigger hypoxia or ATP depletion, which leads to cytochrome c release from mitochondria, activation of caspase-9, and finally results in apoptosis [92]. Therefore, the selective elimination of dysfunctional mitochondria by mitophagy can act as a point mechanism for inhibition of apoptosis by autophagy in both TSCI and IRSCI. This is a meaningful direction requiring further study in the future.

## 6. Pharmacological Intervention of Autophagy in SCI

Autophagy is thought to have a neuroprotective effect in neonatal hypoxia-ischemia-induced brain injury and closed head injury [93,94]. On the other hand, autophagy may play a role in neurodegeneration through autophagic cell death in models of focal cerebral ischemia and neonatal cerebral ischemia [95,96]. To promote the neuroprotection and reduce the detrimental effect, mainly two methods of pharmacological intervention of autophagy after experimental SCI were explored. One evaluates the change of autophagy after the treatment of effective drug for experimental SCI therapy. The other method tests the therapeutic outcome of a drug that directly modulates autophagic function after experimental SCI.

### 6.1. Therapeutic Agent Effect on Autophagy in SCI

Investigating changes in autophagy activity after administration of therapeutic agent believed to lead to beneficial outcomes can provide indirect evidence for autophagy function in experimental SCI. In these studies, it was believed that upregulation of autophagy after administration of these therapeutic agents supports the hypothesis that autophagy exerts a protective effect [97], and the downregulation of autophagy supports the contrary [73]. However, both upregulation and downregulation of autophagy in SCI were found after administration of various therapeutic agents (Table 1).

In compression injury, the therapeutic agents calcitriol (a biologically active metabolite of vitamin D) [102], and metformin (a hypoglycemic agent for the therapy of type 2 diabetes mellitus through adenosine monophosphate-activated protein kinase (AMKP) activation) [103], were found to increase the activity of autophagy in SCI. Specifically, calcitriol promoted locomotor recovery after SCI by reducing oxidative stress and inhibition of apoptosis, and increased autophagy activity [77]. Metformin administration was found to provide neuroprotection for SCI by decreasing lesion issue damage and neurons apoptosis, and increasing the level of autophagy [38]. Interestingly, basic fibroblast growth factor, a member of the fibroblast growth factors [28] and estradiol (a17β-estradiol, E2) [73] were reported to reduce the level of autophagy and promote neuronal survival after spinal cord compression injury.

In contusion injury, the therapeutic agents of atorvastatin and simvastatin (two inhibitors of 3-hydroxy-3-methylglutaryl-coenzyme A reductase) [53,98], metformin [100], exendin-4 (an agonist of glucagon-like peptide-1) [55] and vitamins C and E (antioxidants) [57], worked through the mechanisms of apoptosis inhibition, combined with upregulation of autophagy after SCI. However, methylprednisolone (a synthetic glucocorticoid hormone), valproic acid (a histone deacetylase (HDA) inhibitor) and systemic bisperoxovanadium (a small-molecule protein tyrosine phosphatase (PTP) inhibitor) also have effects of downregulating the level of autophagy in the treatment of contusion injury [47,54,99].

In hemisection injury, granulocyte colony-stimulating factor, a low molecular weight glycoprotein, was found to increase autophagy activity and provide a therapeutic intervention in SCI via inhibiting neuronal apoptosis [97]. The active metabolite of vitamin A, retinoic acid, inhibits endoplasmic reticulum-stress (ER-stress) dependent apoptosis, prevents disruption of the blood-spinal cord barrier, and upregulates autophagy after hemisection injury as well [75]. In I/R injury, hydrogen sulfide, a novel gaseous mediator, promoted behavior functional recovery and activated autophagy.

In summary, either enhancement or inhibition of autophagy was reported after administration of various therapeutic agents even when the same SCI model was used. These mixed results lead to an ambiguous definition of the role of autophagy after SCI, however, validating overall effects of autophagy by administering therapeutic agents and analyzing results has limitations. Therapeutic agents often were not intended to directly target the autophagy pathways, and may instead act on a different pathway which may lead to a feedback regulation of autophagy. Therapeutics that act as agonists or inhibitors in the autophagy pathways would be much more suitable to validate the overall effect of autophagy following SCI.

### 6.2. Agents Directly Modulate Autophagy in SCI

As shown in figure 2, rapamycin specifically inhibits mTOR pathway and increases the formation of autophagosomes, and therefore stimulates the process of autophagy [27]. Hence, this agent was commonly used as an autophagic agonist. The compound 3-methyladenine (3-MA) blocks class III phosphoinositide 3-kinase (PI3K) and inhibits the formation of autophagosomes, efficiently maintaining the balance between synthesis and degradation of intracellular proteins [104] (Figure 2). Therefore, 3-MA is widely used as a powerful inhibitor of autophagy. In these studies of autophagy in SCI, both rapamycin and 3-MA were used to define the role of autophagy. Both protective and detrimental functions of autophagy in SCI have been reported with rapamycin treatment. On one hand, the behavior improvement (the significant increase in Basso, Beattie, and Bresnahan (BBB) testing scores) and mitigation of histological destruction (the reduction of spinal cord cavity, suppression of the motor neuron loss, and inhibition of demyelination) strongly supports the benefit of autophagy in TSCI [26,27,44,63]. Its intrinsic biochemical mechanism is considered to be involved in anti-inflammation [56,58], anti-apoptosis [26,27], suppression of astrocyte proliferation [101], promotion of microtubule stabilization, and axon regeneration [105]. However, autophagy stimulated by rapamycin was also found to suppress ubiquitinated protein clearance and inhibit functional recovery in a rat model of spinal cord compression injury [28]. If assuming no difference among different research setting, the different ways how rapamycin is administered in SCI rodents led to different outcomes in neuronal recovery. Rapamycin solution injected subcutaneously near the wound at a dose of 4 mg/kg/day for 7 days resulted in detrimental outcomes after SCI [28]. However, the dose of 1 mg/kg/day injected intraperitoneally for 7 days after SCI was associated with neuroprotection [26]. In essence, a higher dose and more directly administrated route of rapamycin tended to accentuate SCI. Autophagy may exogenously be overstimulated by the high dose and direct injection route of rapamycin. It is widely accepted that moderate autophagy is involved not only in maintaining cellular homeostasis by degrading and recycling damaged organelles and unwanted proteins but that excessive autophagy may also stimulate cell death via the excessive self-digestion and degradation of essential cellular constituents [63,64]. We believe that the activated autophagy after TSCI provides beneficial functions, and these effects can be magnified in moderate stimulation of autophagy which does not cause excessive upregulation. Whether high dose and direct injection of rapamycin is prone to overstimulate autophagy in TSCI still requires further research.

While stimulating autophagy with rapamycin may lead to detrimental outcomes due to overstimulation of autophagy, 3-MA, an inhibitor of autophagy, can also be used to study the effect of autophagy on SCI without the risk of overstimulation. The role of 3-MA in SCI has only been investigated in the hemisection injury and IRSCI model. In hemisection injury, 3-MA injection 4 h after SCI (2.5 mg/kg, intraperitoneal (ip)) resulted in declined BBB testing score and upregulation of apoptosis [27]. These results indicate that autophagy is beneficial for recovery after hemisection injury. In IRSCI, 3-MA administration immediately after spinal cord ischemia (2.5 mg/kg, ip) decreased BBB testing scores, enhanced motor neurons loss, and promoted cell apoptosis in rats [63]. However, for 3-MA administrated at 24 h after injury (2.5 mg/kg, ip), outcomes were opposite, with increased BBB, improved neuron survival, and reduced apoptosis [63]. These results suggest that at different stages in IRSCI, autophagy play different roles. Autophagy provides a neuroprotective function at the early stage, and a detrimental effect at the later stage after IRSCI. The effect of 3-MA in compression and contusion injury remains unknown and to be studied in future research.

According to these results, the effect of autophagy was different in TSCI and IRSCI. In TSCI, autophagy plays a beneficial role for functional recovery after hemisection injury, and may have the same role in compression injury and contusion injury. Whereas in IRSCI, autophagy plays an inconsistent role at different stages; it is protective at the early stage, and detrimental at the later stage. In addition, the exogenous stimulation of autophagy does not always induce a neuroprotective effect in SCI. The reason may potentially be that different extents of exogenous stimulation of autophagy contribute to the inconsistent outcomes after SCI. Autophagy may have a beneficial effect of inhibiting cell death with moderate stimulation of autophagy after SCI, and an opposite worsening effect of inducing cell death in the setting of excessive upregulation. How to define the extent of the exogenous stimulation of autophagy in SCI is a key point that requires further investigation.

## 7. Conclusions

Autophagy plays a complicated role in SCI. Types of SCI and severity may contribute to the differences in outcomes regarding temporal pattern, activation, and function of autophagy. The situation of autophagic flux may also depend on the injury severity in SCI, where severe injury results in the blockade of flux, and moderate injury causes the enhancement of flux after SCI. Autophagy also plays a beneficial role for functional recovery after hemisection injury, and may act with the same role in compression injury and contusion injury. In IRSCI, autophagy is protective at the early stage, but detrimental at the later stage.

Much work remains to further define the roles of autophagy after SCI in regards to temporal pattern, flux, and function. Definition of autophagy involves not only autophagosomes, but also the process of flux. Methods for monitoring autophagic flux, such as testing LC3-II turnover in in the presence and absence of lysosomal degradation inhibitors (baflomycin A1 [106], and chloroquine [107]), and assay of degradation of p62, need to be applied. To accurately understand the function of autophagy after SCI, transgenic animals with autophagic defects, such as Atg5^−/−^ mice, should be used in future studies. Furthermore, SCI may be primarily an inflammatory phenomenon [108]. Literature suggests a pathologic link between inflammation and the process of autophagy in CNS disease [109], however, there is a lack of similar studies in SCI. It is important to explore the crosstalk between autophagy and inflammatory responses after SCI. Finally, the optimized exogenous stimulation of autophagy to promote recovery after SCI needs to be further characterized.

Currently, autophagy-related agents have not been applied in the clinical realm. Nevertheless, autophagy is still a potential therapeutic target. With future development of specific strategies for autophagy intervention in different SCI types, we are hopeful that research in this field will be able to improve patient functional recovery after SCI.

## Figures and Tables

**Table 1:** Therapeutic agents regulate the level of autophagy in experimental SCI.

Agents	Classification	Autophagic Mechanism	Autophagic Regulation	Autophagosomes	Flux	Pathologic Mechanism	Behavior Test	Models
Vitamins C and E [57]	Antioxidants	Not referred	Upregulation	LC3II↑	Not referred	Oxidative stress↓ Apoptosis↓	Hindlimb function↑	Contusion injury (T9–T10)
Exendin-4 [55]	Glucagon-like peptide-1 agonist	Not referred	Upregulation	LC3II/I↑ Beclin1↑	Not referred	Neuron loss↓ Cavity formation↓ Apoptosis↓	Hindlimb function↑	Contusion injury (T9–T11)
Simvastation [53]	Inhibitor of 3-hydroxy-3-methylglutaryl-coenzyme A reductase	mammalian target of rapamycin (mTOR) inhibition	Upregulation	LC3II↑ Beclin1↑	Not referred	Brain-derived neurotrophic factor (BDNF)↓ Glial cell line-derived neurotrophic factor (GDNF)↓ Apoptosis↓	Hindlimb function↑	Contusion injury (T9–T10)
Atorvastatin [98]	Inhibitor of 3-hydroxy-3-methylglutaryl-coenzyme A reductase	Not referred	Upregulation	LC3II↑ Beclin1↑	Not referred	Apoptosis↓	Hindlimb function↑	Contusion injury (T9–T10)
Systemic bisperoxovanadium [54]	Small-molecule protein tyrosine phosphatase (PTP) inhibitor	mTOR activation	Downregulation	LC3II/I↓	Not referred	Neuron loss↓Cavity formation↓	Forelimb function↑	Contusion injury (C5)
Valproic acid [51]	Histone deacetylase (HDA) inhibitor	Not referred	Downregulation	LC3II↓ Beclin1↓	Not referred	Neuron loss↓ Demyelination↓	Hindlimb function↑	Contusion injury (T10)
Methylprednisolone [99]	Synthetic glucocorticoid hormone	Not referred	Downregulation	LC3II↓	Not referred	Apoptosis↓	Hindlimb function↑	Contusion injury (T9)
Calcitriol [77]	Biologically active metabolite of vitamin D	Not referred	Upregulation	LC3II↑ Beclin1↑	Enhancement p62↓	Neuron loss↓ Cavity formation↓ Apoptosis↓	Hindlimb function↑	Compression injury (T9)
Metformin [38,100]	Hypoglycemic agent for the therapy of type 2 diabetes mellitus	Adenosine monophosphate-activated protein kinase (AMPK) actiavation	Upregulation	LC3II↑ Beclin1↑	Enhancement p62↓	Neuron loss↓ Cavity formation↓ Apoptosis↓	Hindlimb function↑	Compression injury (T9); Contusion injury (T9–T10)
Basic fibroblast growth factor (bFGF) [28]	Member of the fibroblast growth factors	mTOR activation	Downregulation	LC3II/I↓	Inhibition p62↑	Neuron loss↓ Cavity formation↓ Ubiquitinated protein↓	Hindlimb function↑	Compression injury (T9)
Estradiol [73]	a17β-estradiol, E2	Not referred	Downregulation	LC3II/I↓ Beclin1↓ Atg5↓ Atg7↓	Inhibition p62↑	Neuron loss↓ Cavity formation↓	Hindlimb function↑	Compression injury (T10)
Granulocyte colony-stimulating factor (G-CSF) [97]	Member of the CSF family of hormone-like glycoproteins	Not referred	Upregulation	LC3II↑	Not referred	Apoptosis↓	Hindlimb function↑	Hemisection injury (T10)
Retinoic acid [75]	Biologically active metabolite of vitamin A	Not referred	Upregulation	LC3II↑	Enhancement p62↓	Apoptosis↓ Disruption of blood-spinal cord barrier↓	Hindlimb function↑	Hemisection injury (T9)
Hydrogen sulfide [42]	Novel gaseous mediator	Micro-RNA-30c inhibition	Upregulation	LC3II↑ Beclin1↑	Not referred	Spinal cord infarction zone↓	Hindlimb function↑	I/R injury (thoracic aorta blocking)
Rapamycin [26,27,56,58,101]	Specifical mTOR agonist	mTOR activation	Upregulation	LC3II↑ Beclin1↑	Enhancement p62↓	Apoptosis↓ Inflammation↓ Neuron loss↓ Cavity formation↓	Hindlimb function↑	Contusion injury (T9–T10); Hemisection injury (T9–T10; T12); Compression injury (T9)

* Whether the effect of rapamycin will be beneficial after SCI remains controversial [27,28]. I/R: ischemia/reperfusion. The symbol, “↑” stands for up-regulation or increase, and “↓” stands for down-regulation or decrease.
